# The role of immune dysregulation in the pathogenesis of type 1 diabetes: a paradigm shift in prevention strategies

**DOI:** 10.17179/excli2025-8233

**Published:** 2025-04-03

**Authors:** Rajiv G. Gopalsamy, Hariharan Govindasamy, Jessiane B. de Souza, Deise M. R. R. Silva, Pedro H. M. Moura, Ronaldy S. Santos, Marina dos S. Barreto, Adriana G. Guimarães, Lucas A. da M. Santana, Lysandro P. Borges, Eloia E. D. Silva

**Affiliations:** 1 Division of Phytochemistry and Drug Design, Rajagiri College of Social Sciences(Autonomous), Kalamassery, Kochi, Kerala 683104, India; 2 School of Sciences, Bharata Mata College (Autonomous), Thrikkakara, Kochi, Kerala 682021, India; 3 Department of Pharmacy, Federal University of Sergipe (UFS), São Cristóvão, Sergipe; 4 Department of Dentistry, Federal University of Sergipe (UFS), Aracaju, Sergipe, Brazil; 5 Department of Biology, Federal University of Sergipe (UFS), São Cristóvão, Sergipe, Brazil

## ⁯⁯⁯

Type 1 diabetes (T1D) is a chronic autoimmune disease caused by the destruction of insulin-producing beta cells in the pancreas by autoreactive T lymphocytes. Classified as a T cell-mediated autoimmune disease (AID), T1D arises from a disrupted balance between T effector cells (Teffs) and T regulatory cells (Tregs), leading to responses against islet-associated self-antigens. Despite accounting for only 10 % of global diabetes cases, T1D incidence has risen by 3-4 % annually over three decades, emphasizing environmental factors (Cobo-Vuilleumier and Gauthier, 2020[2]).

T1D begins with autoantibodies (AAbs) against insulin, such as GAD65, IA-2, and ZnT8, involving immune, environmental, and genetic factors within the islets of Langerhans. Adaptive immune signaling malfunctions activate self-reactive T cells, triggering beta cell destruction. Alterations in neutrophils, natural killer cells, and macrophages increase susceptibility to inflammation, infections, and complications (Vaibhav et al., 2024[11]). T1D patients face heightened risks for autoimmune diseases like Hashimoto's thyroiditis, vitiligo, and celiac disease (Cai et al., 2021[1]).

Innate immunity also initiates T1D, with toll-like receptor (TLR) signaling and inflammation playing significant roles in insulitis. While medical advancements exist, no robust therapy currently prevents beta cell damage or restores insulin production.

T1D pathogenesis stems from genetic predisposition, environmental triggers, and immune dysfunction. Strongly linked to HLA class II alleles, it involves immune responses to beta cell antigens. Environmental factors, such as viral infections and microbiome dysbiosis, further tip the balance toward autoimmunity. Progression includes genetic susceptibility, islet AAbs emergence, and symptomatic hyperglycemia (James et al., 2023[7]; Houeiss et al., 2022[6]). Early stages feature glucose intolerance, while stage 3 includes symptoms like frequent urination and, in severe cases, diabetic ketoacidosis (DKA) (Sims et al., 2021[10]; Draznin et al., 2022[4]).

Key drivers include defective Tregs that fail to suppress autoreactive T cells, elevated pro-inflammatory cytokines (e.g., IFN-γ, IL-1β), and molecular mimicry where viral peptides resemble beta cell antigens (Goswami et al., 2022[5]; Lemos et al., 2024[8]). Emerging therapies, such as antigen-specific immunotherapies and monoclonal antibodies like teplizumab, aim to delay disease onset by modulating immune responses. For instance, teplizumab extended T1D onset by two years in high-risk individuals (Ludvigsson, 2021[9]; Collier, 2024[3]).

Lifestyle interventions, including dietary adjustments and microbiome modulation, show potential in reducing inflammation and promoting immune tolerance (Zhao et al., 2023[12]). Despite progress, the heterogeneity of immune responses necessitates precision medicine approaches. A paradigm shift in T1D prevention involves stage-specific therapies targeting immune dysfunction and beta cell resilience. Advances in immunological research are key to reshaping disease outcomes, offering hope for sparing future generations from T1D complications.

## Notes

Rajiv G. Gopalsamy, Hariharan Govindasamy, and Eloia E. D. Silva contributed equally to this work.

Lysandro P. Borges and Eloia E. D. Silva (Department of Biology, Federal University of Sergipe (UFS), Av. Marcelo Déda Chagas, s/n, Rosa Elze, Zip Code: 49100000, São Cristóvão, Sergipe, Brazil; E-mail: eloiaemanuelly@gmail.com) contributed equally as corresponding author.

## Conflict of interest

None to declare.
